# Effects of Different Re-referencing Methods on Spontaneously Generated Ear-EEG

**DOI:** 10.3389/fnins.2019.00822

**Published:** 2019-08-07

**Authors:** Soo-In Choi, Han-Jeong Hwang

**Affiliations:** Department of Medical IT Convergence Engineering, Kumoh National Institute of Technology, Gumi, South Korea

**Keywords:** electroencephalography (EEG), ear-EEG, re-referencing, mental task classification, brain-computer interface (BCI)

## Abstract

In recent years, electroencephalography (EEG) measured around the ears, called ear-EEG, has been introduced to develop unobtrusive and ambulatory EEG-based applications. When measuring ear-EEGs, the availability of a reference site is restricted due to the miniaturized device structure, and therefore a reference electrode is generally placed near the recording electrodes. As the electrical brain activity recorded at a reference electrode closely placed to recording electrodes may significantly cancel or influence the brain activity recorded by the recording electrodes, an appropriate re-referencing method is often required to mitigate the impact of the reference brain activity. In this study, therefore, we systematically investigated the impact of different re-referencing methods on ear-EEGs spontaneously generated from endogenous paradigms. To this end, we used two ear-EEG datasets recorded behind both ears while subjects performed an alpha modulation task [eyes-closed (EC) and eyes-open (EO)] and two mental tasks [mental arithmetic (MA) and mental singing (MS)]. The measured ear-EEGs were independently re-referenced using five different methods: (i) all-mean, (ii) contralateral-mean, (iii) ipsilateral-mean, (iv) contralateral-bipolar, and (v) ipsilateral-bipolar. We investigated the changes in alpha power during EO and EC tasks, as well as event-related (de) synchronization (ERD/ERS) during MA and MS. To evaluate the effects of re-referencing methods on ear-EEGs, we estimated the signal-to-noise ratios (SNRs) of the two ear-EEG datasets, and assessed the classification performance of the two mental tasks (MA vs. MS). Overall patterns of changes in alpha power and ERD/ERS were similar among the five re-referencing methods, but the contralateral-mean method showed statistically higher SNRs than did the other methods for both ear-EEG datasets, except in the contralateral-bipolar method for the two mental tasks. In concordance with the SNR results, classification performance was also statistically higher for the contralateral-mean method than it was for the other re-referencing methods. The results suggest that employing contralateral mean information can be an efficient way to re-reference spontaneously generated ear-EEGs, thereby maximizing the reliability of ear-EEG-based applications in endogenous paradigms.

## Introduction

Various neuroimaging modalities have been used to explore brain functions and develop brain applications, such as electroencephalography (EEG), magnetoencephalography (MEG), near-infrared spectroscopy (NIRS), functional magnetic resonance imaging (fMRI), transcranial Doppler (TCD), and so on. Each of them has its own advantages and disadvantages in terms of temporal and spatial resolution, portability, and price. Among them, EEG has been widely used to investigate neural substrates of brain functions and develop clinical and real-life applications owing to high temporal resolution, high portability, and reasonable cost (Hwang et al., 2013).

To precisely investigate brain functions, the use of a high-density EEG system is required; EEGs measured from at least 30 scalp sites allow for both cortical-level analysis (source imaging) and sensor-level analysis (Michel et al., 2004). In contrast, the use of a minimum number of electrodes is required without a significant performance drop for developing practical EEG-based applications. Further issues to be considered in this regard include the use of gel for accurate EEG measurement and the need for relatively bulky system consisting of an amplifier, cap, and electrodes. In recent years, a novel EEG system that measures brain activity around the ears, called ear-EEG, was proposed in order to overcome the existing limitations, thereby enabling the development of practical EEG-based applications (Kidmose et al., 2012, 2013a,b; Looney et al., 2012; Do Valle et al., 2014; Bleichner et al., 2015, 2016; Debener et al., 2015; Mikkelsen et al., 2015; Norton et al., 2015; Fiedler et al., 2016, 2017; Goverdovsky et al., 2016, 2017; Zibrandtsen et al., 2016, 2017; Bech Christensen et al., 2017; Gu et al., 2017; Pacharra et al., 2017; Nakamura et al., 2018).

Two different types of ear-EEG systems have been introduced, depending on from where EEG is measured: (i) inside the ears (Looney et al., 2011, 2012; Kidmose et al., 2012, 2013a,b; Mikkelsen et al., 2015; Goverdovsky et al., 2016, 2017; Kappel et al., 2017; Zibrandtsen et al., 2017; Hong et al., 2018; Nakamura et al., 2018) and (ii) behind the ears (Debener et al., 2015; Bleichner et al., 2016; Goverdovsky et al., 2016; Mirkovic et al., 2016; Bleichner and Debener, 2017; Pacharra et al., 2017). The feasibility of ear-EEG to develop brain applications has been verified in terms of set-up time, performance, and long-term use; its set-up time is within several minutes (Looney et al., 2012; Bleichner et al., 2015, 2016; Debener et al., 2015; Goverdovsky et al., 2016; Zibrandtsen et al., 2016), its performance is comparable to that of conventional scalp-EEG (Kidmose et al., 2012, 2013b; Bleichner et al., 2015, 2016; Mikkelsen et al., 2015; Goverdovsky et al., 2016; Mirkovic et al., 2016; Zibrandtsen et al., 2016, 2017; Kappel et al., 2017; Pacharra et al., 2017; Choi et al., 2018), and its performance is maintained across multiple days (Norton et al., 2015). In early studies based on ear-EEG, resting-state EEG has been used to demonstrate its feasibility, where changes in alpha activity were investigated during eyes-closed (EC) and eyes-open (EO) conditions (Looney et al., 2011, 2012; Debener et al., 2015; Mikkelsen et al., 2015; Norton et al., 2015; Bleichner and Debener, 2017; Goverdovsky et al., 2017). One of the primary applications developed based on ear-EEG is brain-computer interface (BCI) (Bleichner et al., 2015, 2016; Debener et al., 2015; Norton et al., 2015; Fiedler et al., 2016, 2017; Mirkovic et al., 2016; Choi et al., 2018; Floriano et al., 2018; Wei et al., 2018), which is a communication channel operated by brain activity for paralyzed patients. Most ear-EEG-based BCIs have been developed based on exogenous paradigms that use EEGs evoked by external stimuli, such as auditory steady-state response (ASSR) (Kidmose et al., 2012, 2013a,b; Looney et al., 2012; Mikkelsen et al., 2015; Norton et al., 2015; Goverdovsky et al., 2016, 2017; Bech Christensen et al., 2017), steady-state visual evoked potential (SSVEP) (Kidmose et al., 2013b; Goverdovsky et al., 2016, 2017), and event-related potential (ERP) (Kidmose et al., 2012, 2013b; Bleichner et al., 2015, 2016; Debener et al., 2015; Norton et al., 2015; Fiedler et al., 2016, 2017; Pacharra et al., 2017). In our recent study (Choi et al., 2018), we verified the feasibility of using ear-EEG to realize an endogenous BCI using two mental tasks [mental arithmetic (MA) vs. mental singing (MS)] that induce high and low cognitive load, respectively. The two mental tasks that induce cognitive workload have been widely used in EEG-based BCI studies (Shin et al., 2016, 2017; So et al., 2017; Choi et al., 2018). Besides the development of BCI applications, ear-EEG has also been used to develop other brain applications, such as seizure detection (Do Valle et al., 2014; Gu et al., 2017; Zibrandtsen et al., 2017, 2018), sleep detection (Zibrandtsen et al., 2016), and brain authentication (Nakamura et al., 2018).

On the other hand, EEGs are measured by calculating the difference in electrical potentials between a reference electrode and recording electrodes. Conventional scalp-EEGs are generally measured with a reference site, such as earlobe, mastoid, or nose because electrical potentials of these sites are relatively inactive in terms of electrical brain activity, and their sites are not too close to most recording electrodes (Junghöfer et al., 1999). However, reference sites can show some variations in electrical potentials (Junghöfer et al., 1999), thereby influencing EEG characteristics measured from other locations. To reduce the impact of reference brain activity, spatial filters have been proposed for re-referencing EEGs, such as common average reference (CAR) (Offner, 1950), Laplacian derivation (Hjorth, 1975), and reference electrode standardization technique (REST) (Yao, 2001). Most previous ear-EEG studies have used specific locations fixed for the reference, which are generally quite close to recording electrodes. In in-ear-EEG systems, four locations have been used as the reference, such as ear lobe (Looney et al., 2011, 2012; Kidmose et al., 2012), helix (Norton et al., 2015; Goverdovsky et al., 2017; Nakamura et al., 2018), mastoid (Zibrandtsen et al., 2016), and one of the recording electrode locations inside the ear (Kidmose et al., 2013b). In behind-ear-EEG systems, one of the electrode locations behind the ear has been typically used as the reference (Debener et al., 2015; Bleichner et al., 2016; Goverdovsky et al., 2016; Mirkovic et al., 2016; Bleichner and Debener, 2017). Some studies have applied re-referencing methods to original ear-EEGs using CAR (Mikkelsen et al., 2017), bipolar configuration on a same ear side (Gu et al., 2017) and an opposite ear side (Gu et al., 2017), and subtraction of the electrical potentials averaged over ipsilateral (Zibrandtsen et al., 2017) and contralateral (Mikkelsen et al., 2015) electrodes. Even though different re-referencing methods have been applied to original ear-EEGs, to the best of our knowledge, no studies have systematically investigated the impact of different re-referencing methods on ear-EEGs.

The objective of this study was to systematically investigate the impact of re-referencing methods on spontaneously modulated ear-EEGs. To this end, we measured two ear-EEG datasets while 18 subjects performed an alpha modulation task (EO and EC) and two mental tasks (MA and MS), and the recorded ear-EEGs were re-referenced with the mentioned five re-referencing methods used in previous ear-EEG studies. The impact of re-referencing methods on spontaneously modulated ear-EEGs was systematically investigated in terms of signal-to-noise ratio (SNR) for both datasets and classification performance for the mental task dataset.

## Materials and Methods

### Subjects

Eighteen individuals voluntarily participated in this study (10 males and 8 females; age range: 21–31 years; mean age: 24.5 ± 2.67 years). None of them had experienced past or present neurological or psychiatric conditions. All subjects provided written informed consent following a detailed explanation of the experiment, and they were monetarily compensated for their participation after the experiment. Three subjects were excluded from data analysis due to excessive fatigue during the experiment and consumption of alcohol in the previous day, which could affect experimental results. The experimental protocol of this study was approved by the Institutional Review Board of Kumoh National Institute of Technology (No. 6250).

### Experimental Design

**FIGURE 1:**
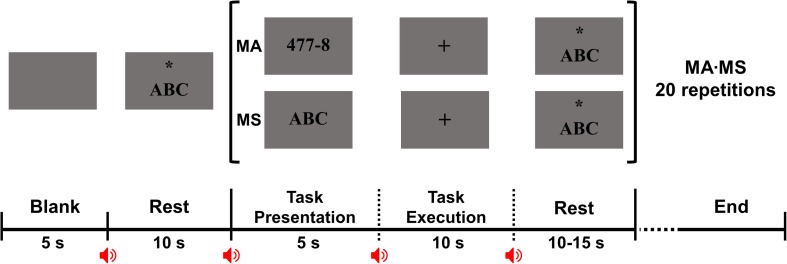
Experimental paradigm used for the two mental tasks. At the beginning of each session, a 10-s rest period is given after presenting a blank screen for 5 s for task preparation. A single trial consists of a 5-s task presentation period, 10-s task execution period, and a 10- to 15-s rest period. In the task presentation period, an instruction indicating either mental task [mental arithmetic (MA) or mental singing (MS)] is presented in which a combination of a single- and three-digit number is presented for MA while the string, “ABC,” for MS. In the task execution period, the subject is asked to perform either MA or MS according to the instruction presented in the task presentation period while focusing on a fixation presented on the center of a monitor to minimize eye movement. The subject is asked to perform sequential subtraction of a single-digit number from a three-digit number for MA, and to internally vocalize the English alphabet song for MS. In the rest period, the string, “ABC” is presented with an asterisk and the subject is asked to sing the English alphabet song while gazing at the asterisk to minimize eye movement. Because MS induces low cognitive load, MS is also used for the rest period, instead of asking the subject not to think of anything.

This study consisted of two different tasks: (i) alpha modulation task and (ii) mental task. In the alpha modulation task, the subjects were asked to alternatively perform EC and EO tasks for 30 s to see the increase in alpha activity during EC as compared to EO, which was repeated six times by each subject. Following this, two different mental tasks were performed by the subjects, i.e., MA and MS. For MA and MS, the subjects were asked to perform sequential subtraction of a single-digit number from a three-digit number and internally sing the English alphabet song, respectively. MA and MS were designed to induce high and low cognitive loads, respectively, thereby aiming to classify two different mental states for BCI purposes (Shin et al., 2017; Choi et al., 2018). Figure 1 shows the experimental paradigm used in this study. A single trial was composed of a 5 s task presentation period, a 10 s task execution period, and a 10 to 15 s rest period. During the task presentation period, a combination of a single- and three-digit number was presented on a monitor for MA, while the string, “ABC,” was presented for MS. Note that 50 different combinations of a single- and three-digit number were prepared for MA to prevent the subjects from becoming accustomed to MA problems. During the task execution period, either task was performed for 10 s according to the instruction. A single session consisted of 20 trials, i.e., 10 MA and 10 MS, and each subject performed five sessions, resulting in 50 trials for each task.

### EEG Data Acquisition

**FIGURE 2:**
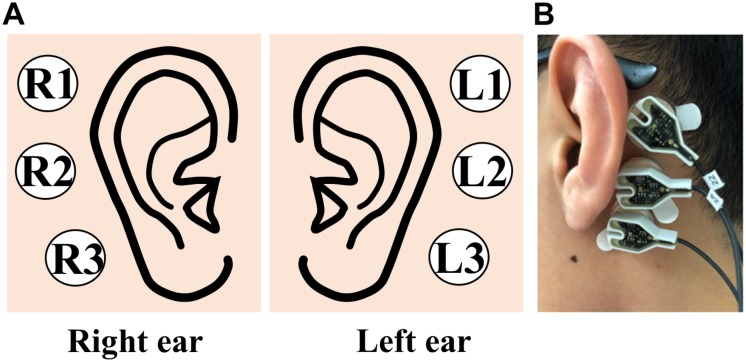
**(A)** Schematic sketch of electrode positions used to record ear-EEGs. **(B)** Picture of three electrodes attached behind the left ear.

The experiment was conducted in a sound-proof room, and the subject was seated in a comfortable armchair in front of a 21-inch monitor (LG, 24MP58VQ, Seoul, South Korea) and binaural speakers (Britz, BR-1000A, Cuve Black2, Paju, South Korea). The monitor and speakers were used to provide the subjects with instructions during the experiment. Even though we measured EEGs from the scalp and behind the ears, we only used EEGs measured behind the ears using six electrodes (three electrodes for each ear) according to the goal of this study (Figure 2A). To mount EEG electrodes behind the ears, a rubber ring holder was attached behind the ear with a double-sided sticker, and an electrode was subsequently inserted into the holder (Figure 2B). The ear-EEG data were measured with the reference and ground electrodes at FCz and Fpz, respectively, according to the international 10–20 system. The measured ear-EEGs were digitized at a sampling rate of 1000 Hz, and impedance was kept below 10 kΩ through the entire experiment. The ear-EEG data used in this study were obtained in our previous study, where we had verified the feasibility of using ear-EEG on the development of an endogenous BCI (Choi et al., 2018).

### EEG Data Analysis

#### Preprocessing and Re-referencing

**FIGURE 3:**
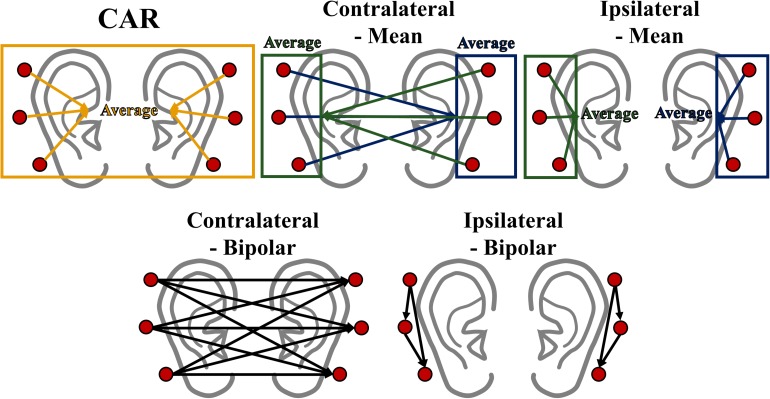
Schematic sketch of the five re-referencing methods used in this study. Note that rectangle and arrow direction indicate average value and re-referencing (subtraction) direction, respectively.

Data analysis was performed using MATLAB (MathWorks, Natick, MA, United States) with EEGLAB (Delorme and Makeig, 2004) and BBCI toolbox (Blankertz et al., 2016). The ear-EEGs were bandpass-filtered between 1 and 50 Hz using a zero-phase fourth-order Butterworth filter, and then down-sampled to 200 Hz. Trials contaminated by eye blinks and body movements were removed based on a peak-to-peak amplitude thresholding method (Choi et al., 2018; Zhang and Lau, 2018), and the number of rejected trials were as follows: 1.27 ± 0.77 for alpha modulation task, 4 ± 1.66 for MA, and 3.66 ± 1.06 for MS. The bandpass-filtered ear-EEGs were then re-referenced to eliminate the effect of the original reference (FCz) as well as to determine the effect of different re-referencing methods on ear-EEGs. It has been well documented that the effect of an original reference is completely eliminated after re-referencing (Dien, 1998; Junghöfer et al., 1999). Five different re-referencing methods were designed using the following: (i) mean of all electrodes (all-mean), (ii) mean of electrical potentials of contralateral electrodes (contralateral-mean), (iii) mean of electrical potentials of ipsilateral electrodes (ipsilateral-mean), (iv) bipolar configuration on contralateral ear sides (contralateral-bipolar), and (v) bipolar configuration on ipsilateral ear sides (ipsilateral-bipolar). The all-mean method subtracts the average value of all six electrodes from the values of each electrode, which was motivated by CAR to remove common noise, although a small number of electrodes (six) were available. The contralateral-mean method subtracts the average value of three electrodes of one side from the values of each electrode on the other side (*e.g.*, L1 – mean of R1, R2, and R3). The contralateral-mean method assumes that common electrical activity of reference electrodes placed on the contralateral side is reduced, thereby providing minimized electrical activity for the reference placed at a relatively long distance from recording electrodes. The ipsilateral-mean method subtracts the average value of three electrodes of one side from the values of each electrode on the same side (*e.g.*, L1 – mean of L1, L2, and L3). This method assumes that common noise of a recording (ipsilateral) side is minimized due to a common average. The contralateral- and ipsilateral-bipolar methods use the difference between two electrodes attached on the opposite and same sides, respectively. We designed two bipolar methods to determine the average effect on re-referencing by comparing with the contralateral- and ipsilateral-mean method. Note that the contralateral-bipolar method created nine re-referenced channels (3 electrodes for the left side × 3 electrodes for the right side) while the other four methods created six re-referenced channels. Each re-referencing method was independently performed for each time point. Figure 3 represents the schematic sketch of the five re-referencing methods.

#### Alpha Modulation Task

To qualitatively see the impact of different re-referencing methods on alpha modulation, time-frequency analysis was performed using short-time Fourier transformation (1-s window size with 90% overlap) for each of EO and EC data. To quantitatively evaluate re-referencing impact on ear-EEGs, spectral amplitudes between 8 and 15 Hz were estimated for each of the re-referenced channels in terms of SNR, and their SNRs were averaged over all subjects for each re-referencing method. Because α-band varied slightly from one subject to the other, we used a broader band of 8–15 Hz by considering different α-bands for each subject. The SNR was calculated as follows:

(1) $$\text{SNR}=10\times \log_{10}\left(\frac{A\,l\,p\,h\,a\,p\,o\,w\,e\,r_{E\,C}}{A\,l\,p\,h\,a\,p\,o\,w\,e\,r_{E\,O}}\right)$$

#### Mental Task (Mental Arithmetic vs. Mental Singing)

Event-related (de)synchronization (ERD/ERS) was calculated to visually inspect changes in EEG characteristics during MA and MS, influenced by re-referencing methods. To this end, epochs from −2 to 10 s based on the task onset of MA and MS were extracted, and baseline correction was performed by subtracting the mean value of the EEG data recorded between −2 and 0 s from each epoched data (Pfurtscheller, 1977). Because stronger ERS is generally observed in θ- and α-bands during MA as compared to MS (see Figures 7–9 in advance), SNR was calculated using ERD/ERS values between 5 and 13 Hz to quantitatively estimate the effect of different re-referencing methods on ERD/ERS using Eq. 1 (MA/MS). A multi-band common spatial pattern (CSP) was applied to the epochs of MA and MS to compute the most discriminative features for classification, where five frequency bands were used: δ-band (1–3 Hz), θ-band (4–7 Hz), α-band (8–13Hz), β-band (14–29 Hz), and γ-band (30–50 Hz) (Ramoser et al., 2000; Lemm et al., 2005). A shrinkage linear discriminant analysis (sLDA) was used as a classifier, and 10 × 10-fold cross-validation was performed to evaluate the performance of classifying MA with MS (Peck and Ness, 1982; Schäfer and Strimmer, 2005).

## Results

### Alpha Power Changes During EC and EO

**FIGURE 4:**
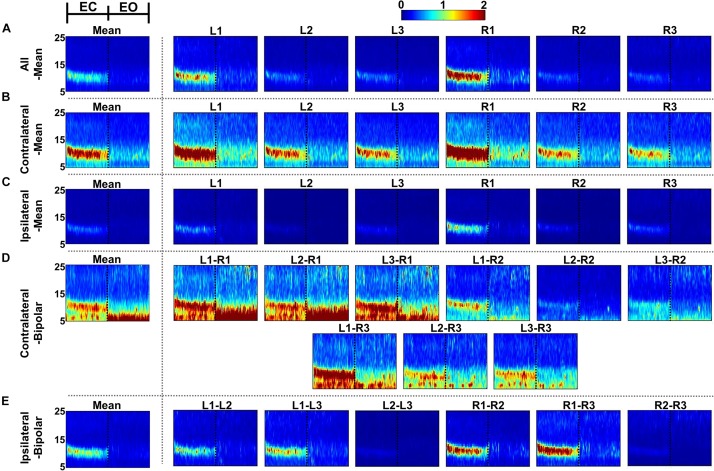
Grand-average time-frequency maps during eyes-closed (EC) and eyes-open (EO) conditions for the five re-referencing methods: **(A)** all-mean, **(B)** contralateral-mean, **(C)** ipsilateral-mean, **(D)** contralateral-bipolar, and **(E)** ipsilateral-bipolar. Grand-average time-frequency maps for each method are presented in the first column, which are denoted by “Mean,” and those for the re-referenced channels of each method are presented on the right side from the second column. The x-axis of each time-frequency map indicates the task period during EC (0–30 s) and EO (30–60 s). A significant increase in alpha activity is clearly observed from most individual re-referenced channels for all re-referencing methods. The contralateral-mean method generally show stronger increase in alpha power during EC, and L1 and R1 channels as well as bipolar channels created using either L1 or R1 also show stronger alpha activity as compared to other channels.

**FIGURE 5:**
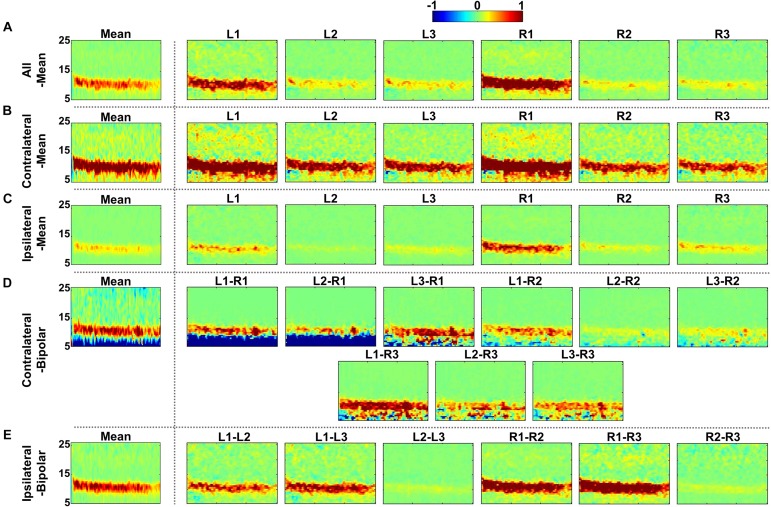
Grand-average time-frequency difference maps between eyes-closed (EC) and eyes-open (EO) for the five re-referencing methods: **(A)** all-mean, **(B)** contralateral-mean, **(C)** ipsilateral-mean, **(D)** contralateral-bipolar, and **(E)** ipsilateral-bipolar. Grand-average time-frequency difference maps for each method are presented in the first column, which are denoted by “Mean,” and those for re-referenced channels of each method are presented on the right side from the second column. A significant increase in alpha power is clearly observed from all individual re-referenced channels for all of the re-referencing methods. The contralateral- mean method generally shows a stronger increase in alpha power during EC, and L1 and R1 channels as well as bipolar channels created using either L1 or R1 also show a stronger increase in alpha power during EC as compared to other channels.

Figures 4, 5 show the time-frequency maps of EC and EO conditions and their differences for the five re-referencing methods, respectively, where grand average results are presented with those of individual re-referenced channels. As expected, alpha power increases during EC as compared to EO regardless of the re-referencing method, resulting in a significant difference in alpha power between EC and EO (Figure 5). Time-frequency analysis of each re-referencing method shows quite similar trends, except that relatively stronger power is observed during both EC and EO at low frequencies (<8 Hz) for the contralateral-bipolar method. The contralateral-mean method generally shows a stronger increase in alpha power during EC (Figure 4B), and therefore the difference in alpha power between EC and EO is also most dominant in the contralateral-mean method (Figure 5B). From the perspective of individual re-referenced channels, L1 and R1 are more sensitive to alpha modulation during EC as compared to other channels; increase in alpha power during EC is most dominant for three mean re-referencing methods (all-, contralateral-, and ipsilateral-mean) at L1 and R1, and a similar trend is observed for two bipolar re-referencing methods when L1 or R1 is employed (Figure 4). This phenomenon also leads to dominant power differences in α-band between EC and EO for L1 and R1 (Figure 5).

**FIGURE 6:**
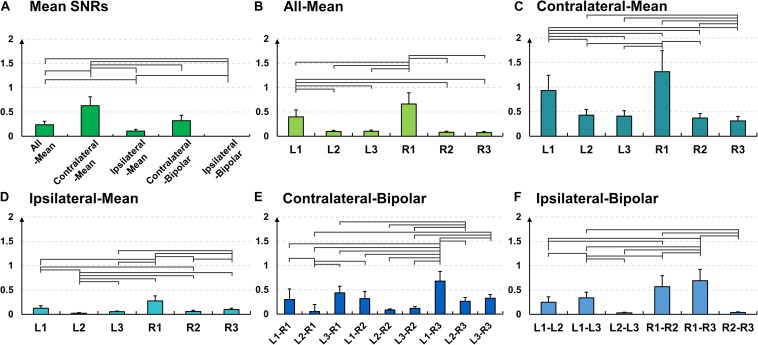
Mean signal-to-noise (SNR) values and their standard errors for the alpha modulation task over **(A)** different re-referencing methods [Friedmann test with Wilcoxon signed-rank sum test: false discovery rate (FDR)-corrected *p* < 0.05]. Mean SNR values and their standard errors for the alpha modulation task over all subjects for each of the five re-referencing methods: **(B)** all-mean, **(C)** contralateral-mean, **(D)** ipsilateral-mean, **(E)** contralateral-bipolar, and **(F)** ipsilateral-bipolar. The mean SNRs of L1 and R1 are significantly higher than other channels for three mean re-referencing methods **(B–D)** for most cases, and those of bipolar channels created using either L1 or R1 also show significantly higher SNRs than do other bipolar channels for most cases **(E,F)**.

The SNR results coincide with the results of time-frequency analysis. The mean SNR of the contralateral-method is statistically higher than those of the other methods (Figure 6A); 0.23 ± 0.07 for all-mean, 0.63 ± 0.18 for contralateral-mean, 0.10 ± 0.03 for ipsilateral-mean, 0.28 ± 0.10 for contralateral-bipolar, and 0.32 ± 0.11 for ipsilateral-bipolar (Friedmann test with Wilcoxon signed-rank sum test: false discovery rate (FDR)-corrected *p* < 0.05). Further, L1 and R1 show statistically higher SNRs than do the other channels in most cases for the three mean re-referencing methods (all-, contralateral-, and ipsilateral-mean) (Figures 6B–D), and the mean SNRs of bipolar channels including either L1 or R1 are generally higher than those of the other bipolar channels (Figures 6E,F).

### ERD/ERS During Mental Tasks

**FIGURE 7:**
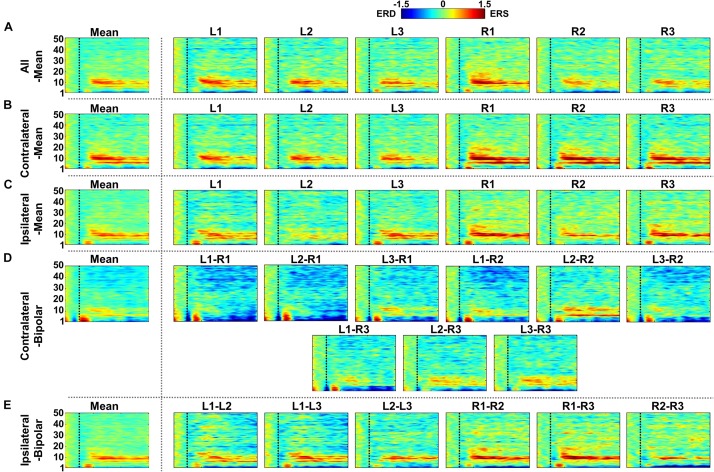
Grand-average event-related (de)synchronization (ERD/ERS) maps during mental arithmetic (MA) for the five different re-referencing methods: **(A)** all-mean, **(B)** contralateral-mean, **(C)** ipsilateral-mean, **(D)** contralateral-bipolar, and **(E)** ipsilateral-bipolar. Grand-average ERD/ERS maps for each method are presented in the first column, which are denoted by “Mean,” and those for re-referenced channels of each method are presented on the right side from the second column. The dashed vertical line in each ERD/ERS map denotes task onset time. Increased ERS is generally observed during MA around θ- and α-bands for all re-referencing methods, while dominant ERD is observed for the contralateral-bipolar method in a relatively high frequency band (>20 Hz). Moreover, right-ear channels re-referenced by the contralateral-mean method show relatively stronger ERS as compared to the other channels re-referenced by other methods.

**FIGURE 8:**
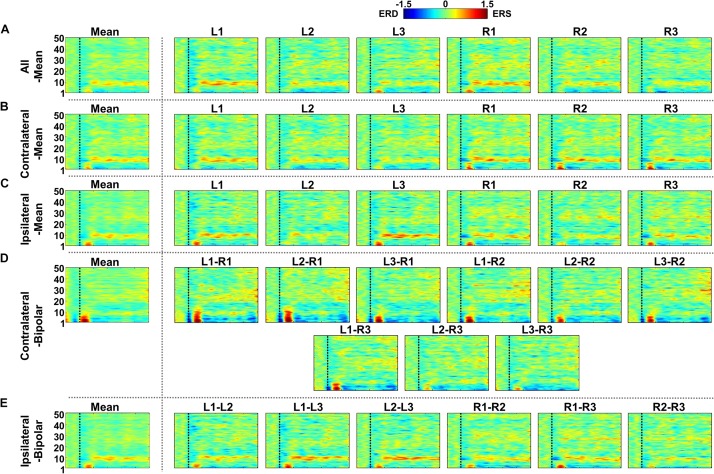
Grand-average event-related (de)synchronization (ERD/ERS) maps during mental singing (MS) for five different re-referencing methods: **(A)** all-mean, **(B)** contralateral-mean, **(C)** ipsilateral-mean, **(D)** contralateral-bipolar, and **(E)** ipsilateral-bipolar. Grand-average ERD/ERS maps for each method are presented in the first column, which are denoted by “Mean,” and those for re-referenced channels of each method are presented on the right side from the second column. The dashed vertical line in each ERD/ERS map denotes task onset time. In general, ERS is observed for most re-referenced channels in α-band, but this effect is weaker than that for mental arithmetic (MA).

**FIGURE 9:**
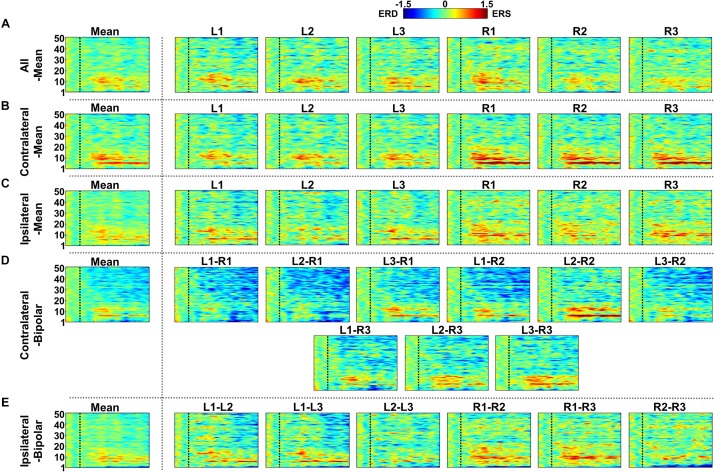
Grand-average event-related (de)synchronization (ERD/ERS) difference maps between mental arithmetic (MA) and mental singing (MS) for five different re-referencing methods: **(A)** all-mean, **(B)** contralateral-mean, **(C)** ipsilateral-mean, **(D)** contralateral-bipolar, and **(E)** ipsilateral-bipolar. Grand-average ERD/ERS difference maps for each method are presented in the first column, which are denoted by “Mean,” and those for the re-referenced channels of each method are presented on the right side from the second column. The dashed vertical line in each ERD/ERS map denotes task onset time. Increased ERS is generally observed around θ- and α-bands for all re-referencing methods, while dominant ERD is observed for the contralateral-bipolar method in a relatively high frequency band (>20 Hz). Right-ear channels re-referenced by the contralateral-mean method show relatively stronger ERS than do the channels re-referenced by other methods.

Figures 7–9 show ERD/ERS maps during MA and MS, as well as their difference (MA–MS), respectively, for each of the five re-referencing methods. In general, dominant ERS (power increase) is observed during MA around θ- and α-bands (Figure 7) while that weakens during MS (Figure 8) regardless of the re-referencing method. The contralateral-bipolar method shows broad ERD in a relatively high frequency band (>20 Hz) during MA (Figure 7D). Also, channels attached to the right ear show stronger ERS as compared to those attached on the left ear, which was most dominant when using the contralateral-mean method (Figure 7). According to the ERD/ERS results, ERD/ERS difference maps (MA–MS) also show significant ERS around θ- and α-bands for all re-referencing methods, as well as ERD over 20 Hz for the contralateral-bipolar method (Figure 9). More ERS differences between MA and MS are observed in the right channels re-referenced by the contralateral-mean method.

**FIGURE 10:**
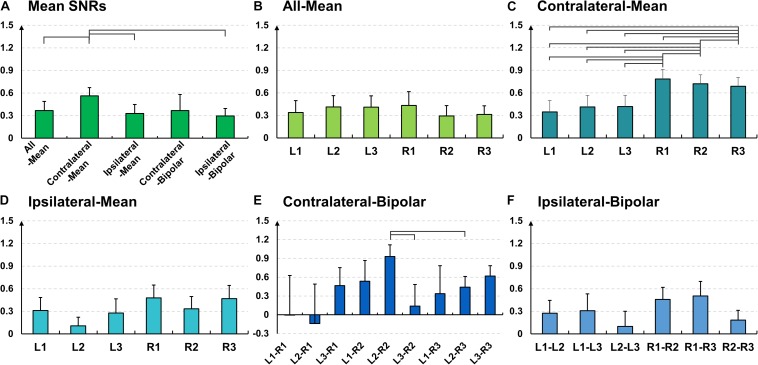
Mean signal-to-noise (SNR) values and their standard errors for the mental task over **(A)** different re-referencing methods (Friedmann test with Wilcoxon signed-rank sum test: false discovery rate (FDR)-corrected *p* < 0.05). Mean SNR values and their standard errors for the mental task over all subjects for each of the five re-referencing methods: **(B)** all-mean, **(C)** contralateral-mean, **(D)** ipsilateral-mean, **(E)** contralateral-bipolar, and **(F)** ipsilateral-bipolar. The right-ear channels re-referenced by the contralateral-mean method show significantly higher SNRs than do the left-ear channels, and they also show higher SNRs than do most other channels re-referenced by different methods, except one contralateral-bipolar channel (L2–R2).

Similar to the SNR result of the alpha modulation task shown in Figure 6, the contralateral-mean method shows the highest mean SNR, being statistically higher than those of the other methods, except the contralateral-bipolar method (Friedmann test with Wilcoxon signed-rank sum test: FDR-corrected *p* < 0.05) (Figure 10A). No significant difference is found in the other cases. On the other hand, unlike the alpha modulation task (EO/EC), L1 and R1 do not show significant differences as compared to the other channels. All re-referenced channels show similar mean SNRs within each of the three re-referencing methods (all-mean, ipsilateral-mean, and ipsilateral-bipolar method), and no statistical difference is found between channels within each method (Figures 10B,D,F). Right-ear channels show significantly higher mean SNRs than do left-ear channels for the contralateral-mean method (Figure 10C), and their mean SNRs are also higher than those of all channels re-referenced by the other four methods, except one contralateral-bipolar channel, L2–R2 (Figure 10E).

### Classification Performance

**FIGURE 11:**
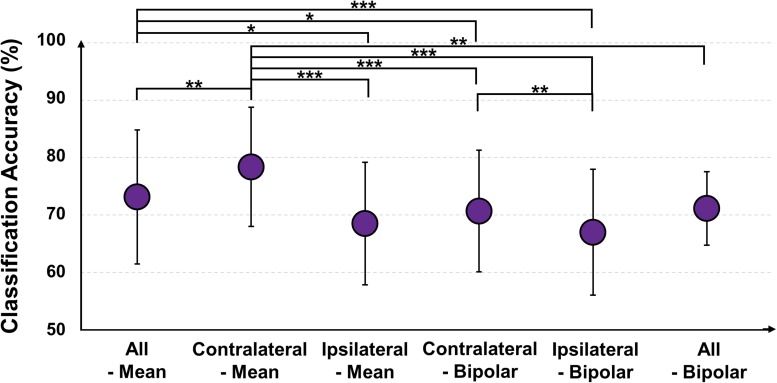
Mean classification accuracies of five re-referencing methods [mental arithmetic vs. mental singing; Friedmann test with Wilcoxon signed-rank sum test: false discovery rate (FDR)-corrected ^∗^*p* < 0.05, ^∗∗^*p* < 0.01, and ^∗∗∗^ *p* < 0.001].

Figure 11 shows the mean classification accuracies of each re-referencing method (MA vs. MS), and those of all-mean, contralateral-mean, ipsilateral-mean, contralateral-bipolar, and ipsilateral-bipolar are 73.13 ± 11.68, 78.37 ± 10.38, 68.52 ± 10.67, 70.68 ± 10.57, 67.00 ± 10.95, respectively. The contralateral-mean method shows the highest accuracy, and its performance is significantly higher than that of all other methods (Friedmann test with Wilcoxon signed-rank sum test: FDR-corrected ^∗^*p* < 0.05, ^∗∗^*p* < 0.01, and ^∗∗∗^ *p* < 0.001). The classification performance of each re-referencing method is almost proportional to the mean SNRs of each re-referencing method shown in Figure 10.

## Discussion

Because EEGs are measured at positions of recording electrodes with respect to a reference electrode, the resulting EEGs are affected by electrical brain activity measured at that reference electrode (Bertrand et al., 1985; Dien, 1998). Thus, re-referencing procedures are often required to mitigate the impact of the reference brain activity (Offner, 1950; Hjorth, 1975; Yao, 2001). Several reference sites, such as mastoid, nose, and earlobe, have been widely used for measuring scalp-EEGs because they are relatively inactive in terms of brain activity as compared to the positions of recording electrodes. However, in ear-EEG data acquisition, reference sites are limited around the ears, where recording electrodes are also placed due to the compact device structure. Thus, the re-referencing of original ear-EEGs was often introduced to reduce the impact of reference brain activity measured close to recording electrodes (Mikkelsen et al., 2015, 2017; Gu et al., 2017; Zibrandtsen et al., 2017). In this study, to systematically investigate the impact of different re-referencing methods on ear-EEGs, we tested five different re-referencing methods that have been used independently in previous ear-EEG studies (Mikkelsen et al., 2015, 2017; Gu et al., 2017; Zibrandtsen et al., 2017). All qualitative and quantitative results showed that the contralateral-mean method is the most efficient for re-referencing ear-EEGs spontaneously generated from two endogenous paradigms based on alpha modulation and mental tasks.

EEGs re-referenced to the contralateral side generally showed higher amplitudes than did those re-referenced to the ipsilateral side (Gu et al., 2017; Zibrandtsen et al., 2017), which was also clearly observed from the grand average and individual channel results in Figure 4. The two contralateral re-referencing methods showed higher spectral amplitudes than did those of two ipsilateral re-referencing methods during EC and EO conditions in the overall frequency band. However, in return, contralateral re-referencing methods also included unwanted information that seems to be artifact in terms of alpha activity, such as high spectral amplitudes in relatively low frequency bands (Figure 4D). These artifact components were somewhat compensated in the contralateral-mean method by using electrical potentials averaged over all contralateral electrodes as the reference (Figure 4B). Thus, the contralateral-mean method showed higher SNRs for both alpha modulation and mental tasks (Figures 6, 10), and resulted in higher performance in classifying the two mental tasks (MA vs. MS) (Figure 11). On the other hand, the all-mean methods imitating CAR did not show distinctive characteristics in terms of both SNR and classification performance.

Two channels, L1 and R1, were most sensitive to EC-related alpha activity, and thus they showed significantly higher SNRs than did other channels for most cases in each of five re-referencing methods (Figures 4–6). This might be because the position of L1 and R1 is slightly closer to the occipital lobe, which plays a key role in increasing alpha power during EC (Figure 2B). Brain activity of L1 and R1 was better captured using the contralateral-mean method, showing the highest mean SNRs over all re-referencing methods (Figures 6B–F). For MA and MS, right-ear channels showed stronger ERS during MA than did left-ear channels (Figure 7), and their brain activity was also better captured using the contralateral-mean method (Figures 7B, 10C). In a previous study (Friedrich et al., 2012), ERS was more widely observed for right temporo-occipital areas than for the left ones during MA. Because ear-EEGs could be mainly influenced by brain activity generated around temporo-occipital areas, the asymmetric ERS result of the previous study might explain stronger ERS for right-ear channels than for left-ear channels. One contralateral-bipolar channel (L2–R2) showed a relatively higher SNR than did the others (Figure 10E), including the right ear channels re-referenced by the contralateral-mean method for the mental tasks. Even though the one channel (L2–R2) created by the contralateral-bipolar method showed the highest SNR for the mental tasks, the mean SNR of the contralateral-mean method was higher than that of the contralateral-bipolar method (Figure 10A). Note that left-ear channels re-referenced by the contralateral-mean method also showed comparable mean SNRs to the channels re-referenced by other methods. Based on the mentioned results, it can be thought that the contralateral-mean method may capture spontaneously generated ear-EEGs better than the other methods. However, it should be noted that an optimal re-referencing method always depends on brain activity of interest, and thus another re-referencing method could be optimal for other paradigms (*e.g*., exogenous paradigms employing external stimuli to induce specific brain activity).

Previous studies have used ear-EEGs for seizure detection, in which they used two different re-referencing methods conceptually corresponding to the ipsilateral- and contralateral-bipolar methods in our study (Gu et al., 2017; Zibrandtsen et al., 2017, 2018). Two studies reported that ear-EEGs re-referenced by a contralateral-bipolar method showed higher amplitudes than did those re-referenced by an ipsilateral-bipolar method (Gu et al., 2017; Zibrandtsen et al., 2017), as replicated in our study. Because one study focused on a qualitative comparison between scalp- and ear-EEGs in terms of seizure activity, there was insufficient quantitative evidence to compare the effect of two re-referencing methods on ear-EEGs (Zibrandtsen et al., 2017). Another study presented quantitative results on the effect of re-referencing methods in terms of EOG, coherence between scalp- and ear-EEG, and seizure detection performance (Gu et al., 2017). A contralateral-bipolar method showed generally better results than did an ipsilateral one for the mentioned factors, but the difference was non-significant. This result is in line with our finding that two bipolar re-referencing methods generally showed similar results in terms of SNR and classification performance for two mental tasks, although there were some variations between individual bipolar channels in terms of SNR. The other study also used two sets of ear-EEG data re-referenced by an ipsilateral- and a contralateral-bipolar method, but because they used ear-EEGs only to determine a seizure-detection threshold, no quantitative comparison was performed between the two re-referencing methods (Zibrandtsen et al., 2018). Because our study presented information related to re-referencing effects on ear-EEGs based on endogenous paradigms which was not explored by previous studies (Gu et al., 2017; Zibrandtsen et al., 2017, 2018), using more re-referencing methods with different paradigms, our results provide additional insight into the existing literature on re-referencing effects in ear-EEGs.

We used EEGs acquired behind both ears to investigate re-referencing effects on ear-EEGs. As the fundamental goal of using ear-EEGs is to provide an unobtrusive and easy-to-use recording solution for measuring brain activity, employing two ears might hinder the fundamental goal due to the need for a complicated hardware setup. Therefore, ultimately, an ear-EEG device employing either ear should be used to develop a practical application, and thus novel re-referencing methods that can be applied to ear-EEGs measured from one ear should be developed. Among the five re-referencing methods investigated in our study, two ipsilateral methods (ipsilateral-mean and ipsilateral-bipolar) can be used for re-referencing when using either ear to measure ear-EEGs. However, the two ipsilateral methods did not show comparable results to the two contralateral methods that cannot be applied when using ear-EEGs recorded from either ear. Thus, it would be an interesting future topic to develop re-referencing methods that could work efficiently for ear-EEGs measured from just one ear.

In summary, we systematically investigated the impact of re-referencing methods on spontaneously modulated ear-EEGs by simultaneously using five re-referencing methods. A contralateral-mean re-referencing method yielded best results in all analyses with enhanced EEG amplitudes and suppressed artifacts. These results were obtained by using the difference of brain activity between the reference channel created using all electrodes on the same side of the ear and its contralateral electrodes. Some dominant channels showed higher SNRs, such as L1 and R1 for the alpha modulation task and right-ear channels for the mental task, but these were also better captured using the contralateral-mean method. Our results indicate that employing contralateral mean information can be an efficient way to re-reference ear-EEGs spontaneously generated in endogenous paradigms, thereby enabling increased reliability of ear-EEG-based applications in endogenous paradigms.

## Data Availability

The datasets for this manuscript are not publicly available because the data have been internally used in our laboratory. Note that the data used in this study was used for our previous article published in another journal, which is mentioned in our manuscript. Requests to access the datasets should be directed to the corresponding author.

## Ethics Statement

The experimental protocol of this study was approved by the Institutional Review Board of Kumoh National Institute of Technology (No. 6250).

## Author Contributions

S-IC and H-JH designed the experiment, performed the data analysis, and wrote the manuscript. S-IC acquired the data.

## Conflict of Interest Statement

The authors declare that the research was conducted in the absence of any commercial or financial relationships that could be construed as a potential conflict of interest.
