# Corrigendum: Effects of Different Re-referencing Methods on Spontaneously Generated Ear-EEG

**DOI:** 10.3389/fnins.2019.00908

**Published:** 2019-08-29

**Authors:** Soo-In Choi, Han-Jeong Hwang

**Affiliations:** Department of Medical IT Convergence Engineering, Kumoh National Institute of Technology, Gumi, South Korea

**Keywords:** electroencephalography (EEG), ear-EEG, re-referencing, mental task classification, brain-computer interface (BCI)

In the original article, we neglected to include the funders Ministry of Trade, Industry and Energy (MOTIE, Korea), Ministry of Science and ICT (MSIT, Korea), and Ministry of Health and Welfare (MOHW, Korea), who provided funding under the Technology Development Program for AI-Bio-Robot-Medicine Convergence (20001650).

The authors apologize for this error and state that this does not change the scientific conclusions of the article in any way. The original article has been updated.

